# Neurons and Microglia; A Sickly-Sweet Duo in Diabetic Pain Neuropathy

**DOI:** 10.3389/fnins.2019.00025

**Published:** 2019-01-31

**Authors:** Trevor Rajchgot, Sini Christine Thomas, Jo-Chiao Wang, Maryam Ahmadi, Mohammad Balood, Théo Crosson, Jenny Pena Dias, Réjean Couture, Audrey Claing, Sébastien Talbot

**Affiliations:** ^1^Département de Pharmacologie et Physiologie, Faculté de Médecine, Université de Montréal, Montréal, QC, Canada; ^2^Graduate Institute of Microbiology, College of Medicine, National Taiwan University, Taipei, Taiwan; ^3^Johns Hopkins University School of Medicine, Division of Endocrinology, Diabetes and Metabolism, Baltimore, MD, United States

**Keywords:** pain, diabetes, neuropathy, neurons, microglia, oxidative stress, hyperglycemia

## Abstract

Diabetes is a common condition characterized by persistent hyperglycemia. High blood sugar primarily affects cells that have a limited capacity to regulate their glucose intake. These cells include capillary endothelial cells in the retina, mesangial cells in the renal glomerulus, Schwann cells, and neurons of the peripheral and central nervous systems. As a result, hyperglycemia leads to largely intractable complications such as retinopathy, nephropathy, hypertension, and neuropathy. Diabetic pain neuropathy is a complex and multifactorial disease that has been associated with poor glycemic control, longer diabetes duration, hypertension, advanced age, smoking status, hypoinsulinemia, and dyslipidemia. While many of the driving factors involved in diabetic pain are still being investigated, they can be broadly classified as either neuron -intrinsic or -extrinsic. In neurons, hyperglycemia impairs the polyol pathway, leading to an overproduction of reactive oxygen species and reactive nitrogen species, an enhanced formation of advanced glycation end products, and a disruption in Na^+^/K^+^ ATPase pump function. In terms of the extrinsic pathway, hyperglycemia leads to the generation of both overactive microglia and microangiopathy. The former incites a feed-forward inflammatory loop that hypersensitizes nociceptor neurons, as observed at the onset of diabetic pain neuropathy. The latter reduces neurons' access to oxygen, glucose and nutrients, prompting reductions in nociceptor terminal expression and losses in sensation, as observed in the later stages of diabetic pain neuropathy. Overall, microglia can be seen as potent and long-lasting amplifiers of nociceptor neuron activity, and may therefore constitute a potential therapeutic target in the treatment of diabetic pain neuropathy.

## Introduction

Pain is defined as an unpleasant sensation triggered by noxious stimuli, inflammation, or damage to the nervous system. It is an evolutionarily-conserved defensive mechanism that prevents excessive tissue damage and preserves homeostasis by generating defensive withdrawal reflexes (Scholz and Woolf, 2002). Nociception is initiated by the detection of mechanical, chemical, or thermal noxious stimuli by specialized ion channel receptors present on sensory neurons (Scholz and Woolf, 2002). The activation of these ion channels triggers an influx of various cations, depolarizing the neurons' membrane potentials, which, in turn, activate voltage-gated sodium channels (Na_V_s). This leads to an influx of sodium, and the subsequent firing of action potentials. In the peripheral nervous system (first order fiber), painful sensations are relayed by small, myelinated Aδ-fibers (fast pain transmission) and unmyelinated C-fibers (slow pain transmission) to the spinal cord (second order fiber) (Tesfaye and Kempler, 2005). These action potentials then trigger defensive reflexes, and travel up to the brain (third order fiber) where pain information is integrated and its emotional perception occurs.

### Chronic Pain

Chronic pain is a highly debilitating condition and it is the most common reasons for visits to health care providers (Scholz and Woolf, 2002). The most incapacitating type of chronic pain is peripheral neuropathic pain. This pain is unique in regard to its constancy, the severity of its symptoms, and its resistance to current pharmacological treatment (Zimmermann, 2001; Woolf, 2004). Neuropathic pain is usually generated by peripheral nerve damage resulting from neuronal or spinal cord injuries, surgery, cancer, infection, or diabetes (Scholz and Woolf, 2002; Woolf, 2004; Tsuda et al., 2005). In pathological states, this pain often persists after the disappearance of its causal stimulus. In some cases, pain can be perceived more severely, a phenomenon known as hyperalgesia, or can be generated by normally innocuous stimuli, a condition known as allodynia. Tactile allodynia originates from afferent Aβ fibers (light touch/pressure transmission) that gain the ability to release pro-inflammatory neuropeptides (SP and CGRP) in the synaptic cleft, and/or the sprouting of these fibers to the dorsal superficial laminae IIb, a zone normally restricted to the projection of C fibers (Woolf et al., 1992; Miki et al., 1998). In the CNS, thalamic higher-order neurons often become hyperexcitable and act as pain generators or amplifiers (Fischer and Waxman, 2010). For example, increasing N-Methyl-D-Aspartate receptor (NMDAR) phosphorylation reduces its endogenous blockade by magnesium, thereby enhancing calcium (Ca^+2^) and sodium (Na^+^) influx. This ultimately promotes the establishment of spinal windup, which is an increase in the excitability of spinal neurons (Haigh and Blake, 2001).

### Sensitization of Sensory Neurons

Most drugs targeted to alleviate neuropathic pain are designed to block neurotransmission, and as such, only bring temporary relief (Ji and Suter, 2007). Neuropathic pain is often accompanied by persistent inflammation, as evidenced by the high levels of oxidative substances (Pabreja et al., 2011), inflammatory cytokines (Pabreja et al., 2011), and mediators (Tsuda et al., 2005) present in the neuronal micro-environment. Unlike classical neurotransmitters, these inflammatory molecules are mainly produced by peripheral immunocytes and central glial cells (Marchand et al., 2005; Tsuda et al., 2005). The transcriptomic data of nociceptor neurons notably revealed the expression of specific receptors for immunoglobulins, cytokines and chemokines (Chiu et al., 2014). This evidences the role of nociceptors in directly detecting and responding to interleukins (IL)-1β (Samad et al., 2001; Binshtok et al., 2008) and IL-6 (Opree and Kress, 2000), activin (TGFβ member) (Zhu et al., 2007), TNF-α (Wagner and Myers, 1996), CCL3 (Zhang et al., 2005), GDNF (Malin et al., 2006), histamine (Shim et al., 2007), kinin (Talbot et al., 2012; De Brito Gariepy et al., 2013), and PGE_2_ (Samad et al., 2001, 2002) released in the context of pain. Nociceptors can also sense IL-5 produced during allergic airway inflammation (Talbot et al., 2015), IL-31 produced during lymphoma-associated itch (Cevikbas et al., 2014), thymic stromal lymphopoietin (TSLP) and IL-4 produced during atopic dermatitis (Wilson et al., 2013; Oetjen et al., 2017), and IL-33 derived from contact with poison ivy (Liu et al., 2016). Additionally, nociceptors can drive IL-23 production during psoriasis (Riol-Blanco et al., 2014). Intracellular kinases and transcription factors downstream of these tyrosine kinase receptors, include PI3K (Pereira et al., 2015), MAP kinases (Ji et al., 2002a), p38 (Ji et al., 2002b), JAK1 (Ludbrook et al., 2016; Oetjen et al., 2017), and STAT3 (Mori et al., 2011) and their activation can lead to pain. Thus, these kinases lead to the post-translational modifications of ion channel transducers or voltage gated sodium channels (Julius, 2013). Nociceptor sensitization is thereby largely due to a decrease in the activation threshold of transient receptor potential vannilloid-1 (TRPV1) or transient receptor potential ankyrin-1 (TRPA1) (Davis et al., 2000; Bautista et al., 2006), and Na_V_1.7, Na_V_1.8, and Na_V_1.9 (Kerr et al., 2001; Nassar et al., 2004). In short, lowering the activation threshold of nociceptors results in pain hypersensitivity. For example, prostaglandin PGE_2_ is a well-known neuron sensitizer (Samad et al., 2001), which partly explains why non-steroidal anti-inflammatory drugs exhibit analgesic effects in inflammatory conditions (Vardeh et al., 2009). Nerve growth factor (NGF) has also been recognized as a major neuronal sensitizer (Ji et al., 2002b), which has led to the development of neutralizing monoclonal anti-NGF antibodies as a treatment for chronic inflammatory pain conditions (Hefti et al., 2006). These changes in hypersensitivity are limited to sites where sensitizing mediators are produced, which are known as zones of primary hyperalgesia. Outside of these zones, pain hypersensitivity usually results from central sensitization, which involves changes in the CNS (Woolf et al., 1992; Woolf, 2007; von Hehn et al., 2012). During inflammation, many sensitizing mediators are likely to be released simultaneously; therefore, the targeted pharmacological blockade of only one of these agents will have a limited effect. Conversely, targeting the sensitized nerve or convergent signaling mediators or enzymes may have broader and more durable effects as to treating inflammatory pain by stopping it at its source (Khoutorsky and Price, 2017).

## Diabetes

Diabetes, derived from the Greek word *diabanein*, means “to pass through,” in reference to the symptomatic excessive urine production observed in patients (Kumar et al., 2005). The term diabetes, without qualification, usually refers to *diabetes mellitus*, which roughly translates to “excessive production of sweet urine,” known clinically as glycosuria (Kumar et al., 2005). According to the World Health Organization, at least 422 million people worldwide suffered from diabetes in 2014, representing 8.5% of the world's adult population (WHO, 2016). In 2012, diabetes was responsible for 1.5 million deaths, and its incidence is increasing by more than 8% per year (WHO, 2016). In some regions, such as in Eastern Mediterranean countries, prevalence is increasing by nearly 14%. The National Diabetes Information Clearinghouse estimates the yearly costs of diabetes to more than $132 billion in the United States. In terms of pathology, diabetes is the result of chronic high blood sugar stemming from either low insulin production, as observed in type 1 diabetes; or to a severe reduction in the response of insulin receptors (IR) to insulin, as observed in type 2 diabetes (Kumar et al., 2005). Chronic hyperglycemia causes the classical symptoms of diabetes, including polyuria (frequent urination), polydipsia and polyphagia (Kumar et al., 2005). While both types of diabetes share similar symptoms, they can be distinguished by measuring endogenous insulin production (Kumar et al., 2005).

### Type 1 Diabetes

Formerly known as juvenile diabetes, type 1 diabetes (T1D) represents approximately 10% of diabetes cases in North America and Europe. There is currently no known preventive measure against type 1 diabetes, which is considered immune-mediated or idiopathic (Kumar et al., 2005). Insulin-dependent diabetes mellitus is characterized by the auto-immune, T-cell mediated (Rother, 2007) destruction of insulin-producing beta cells of the pancreatic islets of Langerhans. The destruction of β cells triggers insulin deficiency, which leads to increases of glucose in the patient's blood and urine. Evidence indicates that type I diabetes is induced by a combination of genetic susceptibility [mutation(s) to *iddm1, drb*1, *dqa*, and *dqb*1 gene locus], environmental factors [diet, vitamin D deficiency (Mathieu et al., 2005)], or exposure to a driving antigen (exposure to wheat protein (Knip and Siljander, 2008), antibody from cow's milk protein (Virtanen et al., 1994). There is no current preventive measure against T1D, which can be highly pathogenic, or even fatal, if left untreated. Emerging treatments such as pancreas (Noguchi, 2010) and islet transplants (Noguchi, 2009) have shown relatively positive outcomes in pre-clinical models, and are currently being studied in clinical trials. However, drawbacks to implantation include the necessity for immunosuppressant administration, which increased susceptibility to infection and cancer, graft rejection of the implanted pancreas/islets, hypoglycemia, and a current lack of suitable donors (Balamurugan et al., 2014).

### Type 2 Diabetes

Type 2 diabetes (T2D), also known as non-insulin dependent diabetes mellitus, is a metabolic disorder characterized by chronic high blood glycemia and insulin receptor resistance, sometimes in combination with relative insulin deficiency (Kumar et al., 2005). This type of diabetes can be initially managed by increasing exercise and dietary modification. It represents almost 90% of Western countries' diabetic populations (Kumar et al., 2005). The onset of T2D is related to genetic and environmental factors. The environmental detrimental factors can include smoking, obesity, diet, alcoholism, low physical activity, high cholesterol, hypertension, metabolic syndrome, and Cushing syndrome (Kumar et al., 2005). In recent years, compelling research and efforts have been made to genetically identify mutant or polymorphic genes that predispose individuals to develop type 2 diabetes. These have been found to include *tcf7l2, ppar*γ*, fto, kcnj11, notch2, wfs1, cdkal1, igf2bp2, slc30a8, jazf1, hhex* (Groop and Lyssenko, 2008; Lyssenko, 2008) and *mody* genes, which themselves can account for up to 5% of T2D cases (Billings and Florez, 2010). Mutations in both human leptin production and the human leptin receptor gene can cause severe obesity and pituitary dysfunction, which can in turn engender T2D (Clement et al., 1998; Wabitsch et al., 2015).

### Complications of Diabetes

The chronic impairment of glucose metabolism associated with both types of diabetes has been associated with severe macrovascular (cardiovascular) disease and microvascular complications including retinopathy, nephropathy and sensory poly-neuropathy (Schemmel et al., 2009). Neuropathy is the most common complication seen in ambulatory care of type 2 diabetes patients (Schemmel et al., 2009). Overall, the aforementioned complications can result in debilitating and/or life-threatening conditions such as renal failure, erectile dysfunction, blindness, macular edema, impaired wound healing, hypertension, obesity, coronary artery disease, cerebrovascular accidents, heart failure, allodynia, hyperalgesia, nerve degeneration, insensitivity, and limb amputation.

### Diabetic Pain Neuropathy

Diabetic pain neuropathy (DPN) is defined as the presence of signs and symptoms of peripheral nerve dysfunction in people with diabetes after having excluded other potential causes (Crofford, 1995). DPN is considered the principal cause of mortality, morbidity (Ziegler, 2008), and amputation (Molines et al., 2010) in diabetic patients, as well as the most common cause of neuropathy (Obrosova, 2009). The prevalence of DPN is thought to be proportional to disease duration and seems to be potentiated by an improper control of blood glycemia (Kumar et al., 2005). Ten percentage of 1-year diabetes patients suffer from neuropathy; this number increases to 50% amongst 25-year diabetes patients. Overall, 30% of diabetic patients suffer from DPN (Guastella and Mick, 2009). Interestingly, 39% of diabetic patients either receive no treatment for their symptoms or remain unmanaged (Daousi et al., 2004). While the prevalence of poorly-managed blood glycemia makes a significant proportion of diabetic patients highly susceptible to developing DPN, glycemic management in clinical care is slowly improving (Aschner et al., 2018). There is emerging evidence that genetic factors may play an important role in DPN pathogenesis (Prabodha et al., 2018).

DPN symptoms include paresthesia, numbness, and burning (Schemmel et al., 2009), which vary in nature and severity depending on the particular subpopulation of neurons being affected (Kumar et al., 2005). Certain patients with DPN do not present any symptoms; however, most report pain and/or loss of function in distal regions such as in their toes, feet, fingers, hands, or arms (Ziegler, 2008). Thus, at the onset of DPN, peripheral nerves often act as pulse generators, maintaining distal terminals of sensory nerve fibers in a state of hyperexcitability (Obrosova, 2009). When these fibers undergo active degeneration or impaired regeneration, they can begin to generate ectopic discharges, which induce positive pain symptoms. Later stages of DPN are characterized by a progressive loss of neuronal fibers, which is associated with a loss of sensation, and can ultimately cause diabetic foot syndrome (Yagihashi et al., 2007). The specific clinical diagnosis of DPN involves both electrophysiological and electromyography testing, respectively, assessing nerve conduction and muscular responses to electric stimulation (Kumar et al., 2005; Guastella and Mick, 2009). The metrics of blood glycemia, arterial pressure, heart rate, muscle force, reflex quality, and sensitivity to spatiotemporal changes can be used to indirectly help diagnose diabetic neuropathy in a more general sense (Guastella and Mick, 2009).

## A Focus on the Molecular Drivers of Diabetic Pain Neuropathy

**Figure 1:**
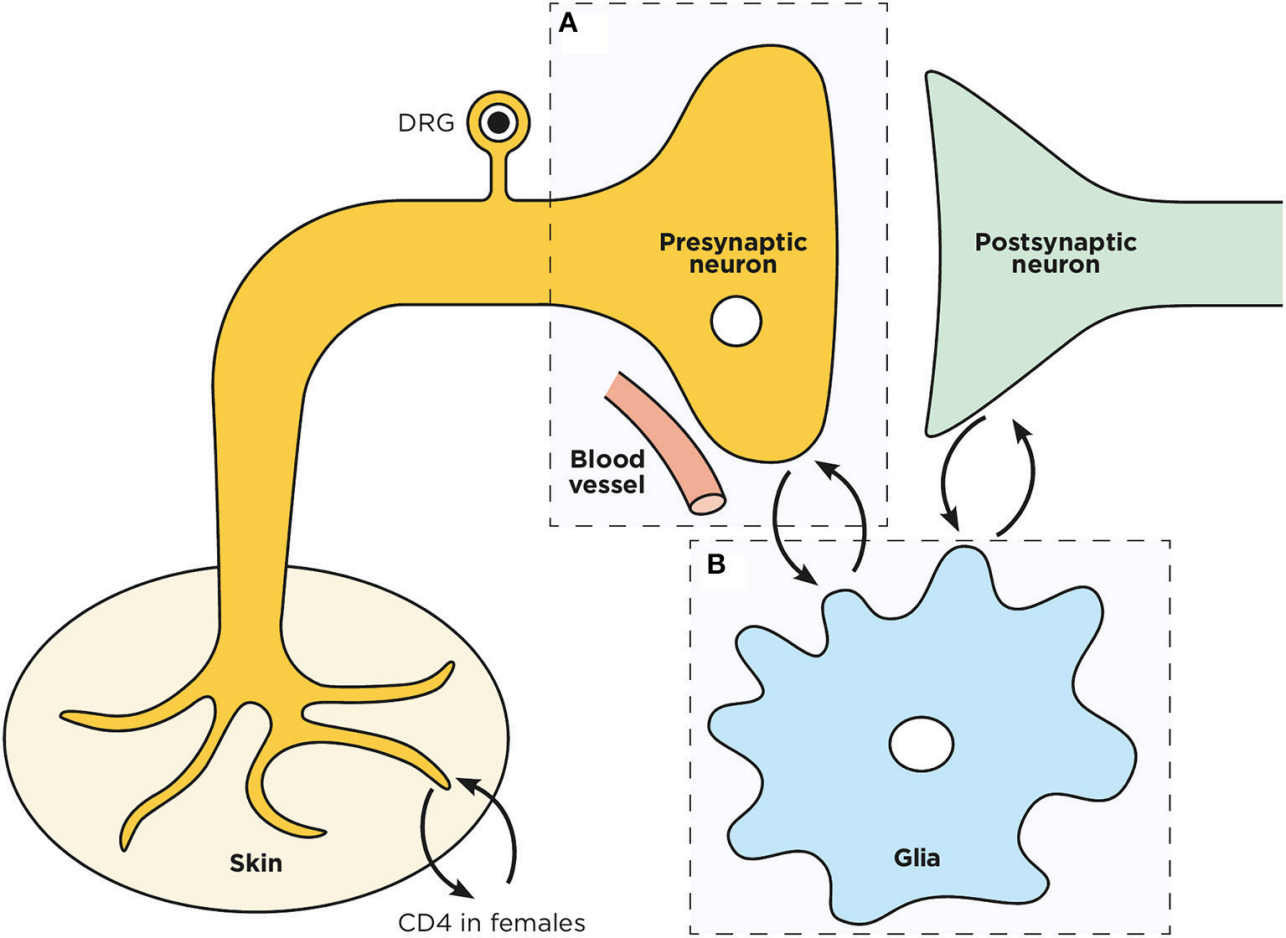
Schematic representation of a spinal dorsal horn tripartite synapse. Overview of the pre **(A)** and post- synaptic neurons interplay with microglia **(B)** in the spinal cord dorsal horn.

**Figure 2:**
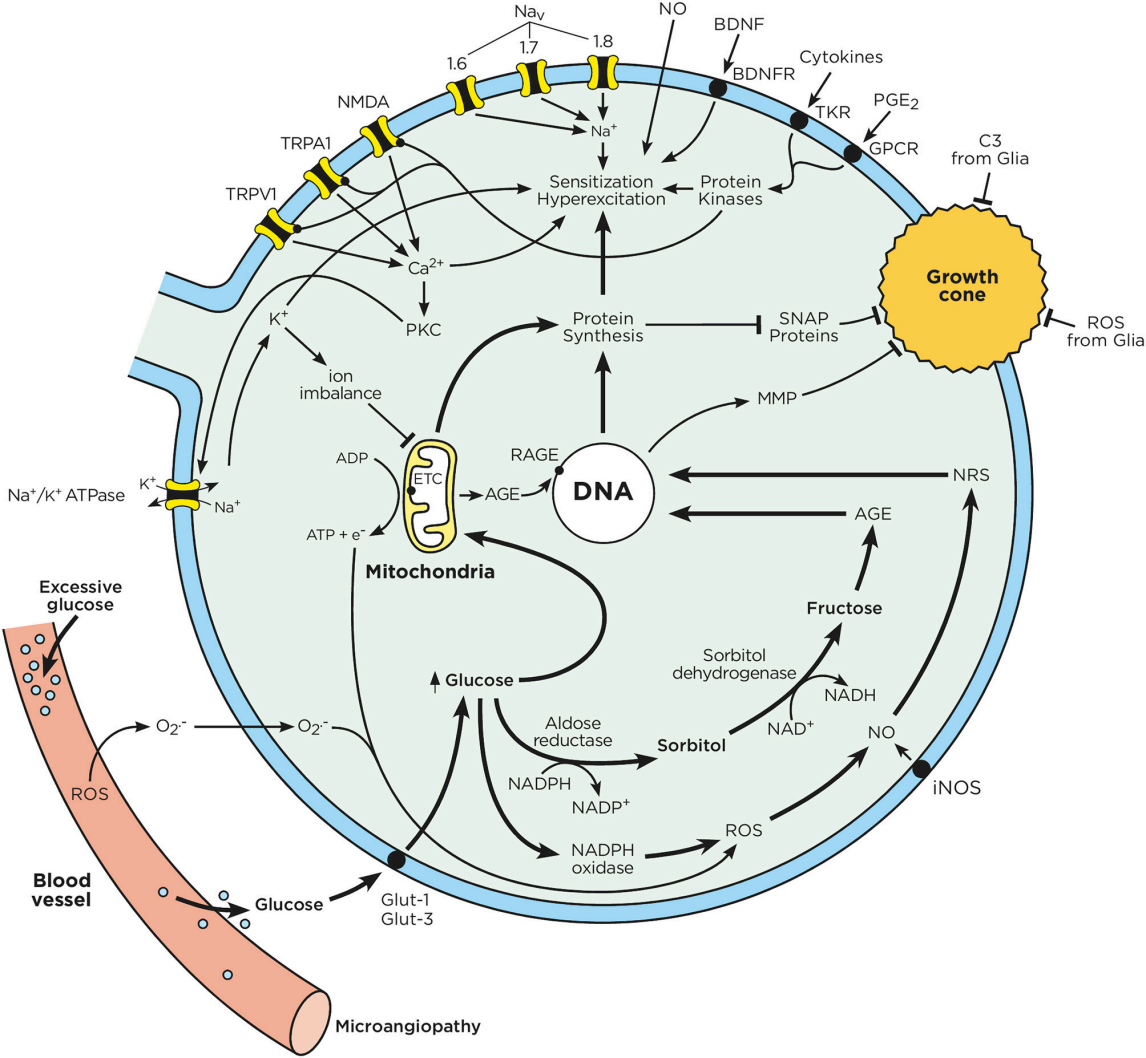
Chronic hyperglycemia impairs neuron function. Sensory neurons have a limited capacity to regulate their uptake of glucose. In the context of chronic hyperglycemia, such as in diabetes, high glucose concentrations drive mitochondria to produce ATP and transfer electrons. Excess glucose is also metabolized through the polyol pathway, leading to the production of advanced glycation end products. The electrons from the mitochondrial respiratory chain combine with intracellular oxygen and nitric oxide to produce ROS and RNS. Consequently, RNS, ROS, and AGE activate nuclear transcription factors, which enhance the expression of ion channel transducers (TRP and Na_V_ channels) in addition to impairing neurons' capacity to self-repair. At the same time, microglia-released mediators (cytokines, ATP, BDNF, NO) stimulate GPCR and tyrosine kinase receptors, triggering downstream signaling cascades, which lead to the phosphorylation of TRP and Na_V_ channels. A decrease in the activation threshold of these ion channel transducers can augment the influx of cations, which ultimately results in action potential firing and ectopic discharges. These effects enhance pain perception and signaling to the CNS. Chronic hyperglycemia also increases oxidative stress in the blood vessels that supply oxygen and nutrients to neuron terminals. This oxidative stress can cause microangiopathy, a phenomenon characterized by the loss of capillaries, which starves neuronal energy supplies. These phenomena are responsible for the loss of neuron terminals and pain insensitivity, as typically observed in later stage of DPN.

**Figure 3:**
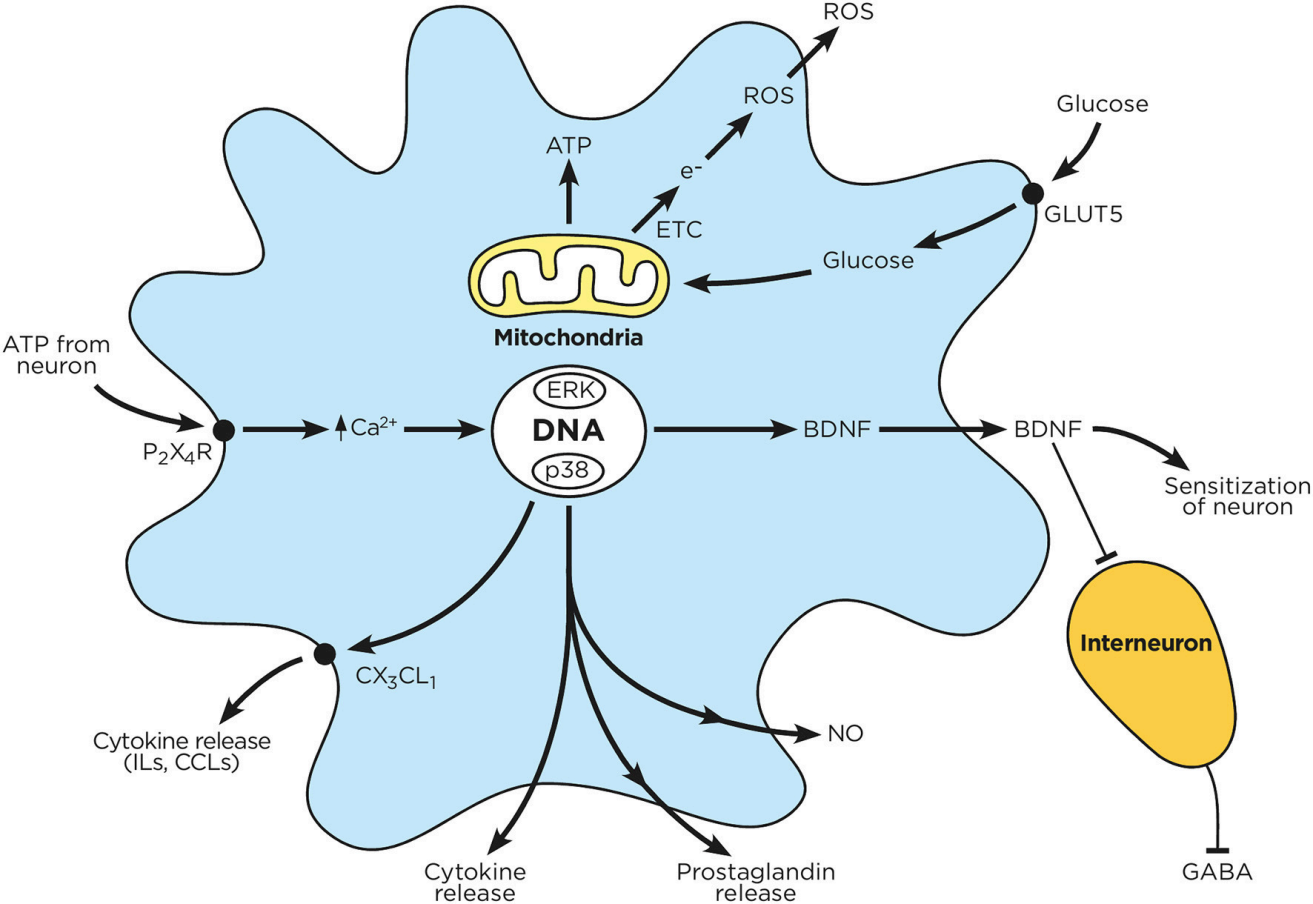
Chronic hyperglycemia impairs microglial function. Circulating glucose is taken up by microglia, which enhances mitochondrial ATP production and electron transfer. The released electrons combine with intracellular oxygen to produce reactive oxygen species. Sensory neurons release ATP, which in turn activates microglial P_2_X_4_R; this drives microglial calcium influx, MAPK activation, receptor phosphorylation and protein transduction (cytokines, prostaglandins, BDNF) as well as NO production. These mediators are subsequently released by microglia, and either block inhibitory interneurons or enhance neuronal activation.

The origins of DPN are multifactorial (Figure 1), and result from neuron intrinsic (Figure 2) and extrinsic factors (Figure 3). This review will examine pre-clinical evidence supporting how chronic hyperglycemia dysregulate neurons' biochemical pathways, activates glia and how such impairments trigger DPN. Current theories (Brownlee, 2001, 2005) regarding neurons intrinsic factor driving the development of DPN include: uncontrolled oxidative stress (section Reactive Oxygen Species) (Nishikawa et al., 2000; Pop-Busui et al., 2006), the formation of reactive nitrogen species (section Reactive Nitrogen Species) (Zochodne and Levy, 2005), the formation of advanced glycation end products (section Advanced Glycation End Products) (Brownlee, 2005; Sugimoto et al., 2008), impaired Na^+^/K^+^ ATPase activity (section Ion Imbalance) (Vague et al., 1997; Gerbi et al., 1998; Raccah, 1998), an imbalance in the polyol pathway (section Polyol Pathway) and/or to the activity of the aldol reductase (section Aldose Reductase) (Oates, 2002). Extrinsically, it is believed that, at the spinal synapse, the neuro-immune interplay occurring between activated microglia and pain-sensing neurons maintains DPN (section Painful Glia to Microglia, an Emerging Target in DPN). The neuronal loss of energy supply occurring through microangiopathy (see Microangiopathy section) appears to be responsible for the loss of sensation observed in later stages of DPN. Finally, future therapeutic avenues will be discussed in the Conclusion and Future Therapeutic Directions section.

## Neuron Intrinsic Factors Driving Diabetic Pain Neuropathy

### Reactive Oxygen Species

Cellular aerobic respiration generates the majority of intracellular free radicals (Kumar et al., 2005; Marieb et al., 2009), which are implicated in normal aging processes (Kumar et al., 2005). Cells are said to be in an oxidative stress state when their levels of reactive oxygen species (ROS) exceed their antioxidant capacity (Kumar et al., 2005). ROS are characterized by their high reactivity stemming from their unpaired valance electrons. These electrons can damage or modify the function of RNA, DNA and proteins. Given that neurons are unable to limit their glucose uptake (Brownlee, 2001), possess numerous mitochondria and long axons make them highly sensitive to oxidative damage. Excessive glucose metabolism by the mitochondrial respiratory chains increases the generation of superoxide anions (Nishikawa et al., 2000).

As seen in Figure 2, mitochondrial superoxide anions reduce glyceraldehyde-3-phosphate dehydrogenase (GAPDH) activity, which in turn reduces cells' anti-oxidative capacity (Du et al., 2000). The exposure of mitochondria to ROS progressively induces mitochondrial dysfunction, which in turn promotes energy deficiency, axonal degeneration and DPN. Mitochondria are key regulators of cell survival and apoptosis. Damaged mitochondria trigger axon degeneration through caspase activation and cycles of fusion and fission (Green and Reed, 1998). The fission of mitochondria is partly regulated by dynamin related protein 1 (DRP1) (Twig et al., 2008). Increased DRP1 levels have been associated with mitochondria dysfunction, reduced ATP production, and axonal degeneration (Leinninger et al., 2006). DRP1 is notably upregulated in the axons of diabetic patients (Leinninger et al., 2006). Overall, an excess of ROS generation along with the inability of neurons to metabolize free radicals can promote the progressive loss of organelles and dysfunction in nuclear cell membranes (Figueroa-Romero et al., 2008).

ROS also reduce axon neurotrophic factor (IGF-1, IGF-II, NGF, and NT-3) production levels, thereby impairing neurons' ability to regenerate (Ishii, 1995; Tomlinson et al., 1997). High glucose levels stimulate the generation of pro-oxidant and highly reactive advanced glycation end products (AGE; section Advanced Glycation End Products) (Baynes and Thorpe, 1999). AGEs and ROS appear to be interdependent (Metz et al., 2003; Monnier, 2003), and central to the etiology of neurovascular dysfunction (Cameron et al., 2001). AGE generation is enhanced by oxygen and ROS; AGE formation can trigger ROS generation and oxidative damage (Monnier, 2003). Finally, hyperglycemia promotes the over-activation of polyol pathways (section Polyol Pathway), reducing cells' NADPH/NADP^+^ ratios and neurons' antioxidant capacities (Figueroa-Romero et al., 2008). Chronic hyperglycemia also enhances PKC activity, either through PLC-DAG pathways, or by reducing DAG-kinase activity (Xia et al., 1994) (King and Loeken, 2004). Enhanced PKC activity also increases mitochondrial NADPH oxidase activity (Inoguchi et al., 2000), further enhancing ROS levels (Balbi et al., 2018). A visual summary of ROS effects on neurons and glia can be found in Figures 2, 3.

ROS are normally metabolized by endogenous antioxidant enzymes such as superoxide dismutase, catalase and glutathione peroxidase, and by certain vitamins such as A, C (ascorbic acid) and E (tocopherol) (Figueroa-Romero et al., 2008; Midaoui et al., 2015; Talbot et al., 2016a). Nutritional supplementation with antioxidants was shown to reduce DPN in rodents (Pop-Busui et al., 2006). While alpha-lipoic acid and superoxide dismutase improved symptoms and electroneurographic parameters among subjects with diabetic neuropathy (Bertolotto and Massone, 2012), clinical studies generally have shown mixed results in terms of antioxidant efficacy (Oyenihi et al., 2015). Overall, it is believed that increasing the bioavailability of antioxidant, as well as associated reductive stress, have limited impact on the patients' health outcome.

### Reactive Nitrogen Species

Nitric oxide (NO) is a potent vasoactive gas formed by three nitric oxide synthase (NOS) isoenzymes: neuronal (nNOS), endothelial (eNOS), and inducible (iNOS) (Kumar et al., 2005). In physiology, eNOS releases NO, which dilates vascular endothelial cells (Kumar et al., 2005) and reduces platelet aggregation (Riddell and Owen, 1999). In the context of inflammation, as is seen in diabetes, iNOS is overexpressed/activated, producing large amounts of NO (~100-fold than other NOS) (Vareniuk et al., 2008; Haddad and Couture, 2016). In DPN, iNOS hyperactivation is found in keratinocytes, macrophages, leukocytes, sensory neurons, and microglia (Zochodne et al., 2000). Excess NO from nNOS acts as a pro-nociceptive mediator in sensory C fibers (Matsui et al., 2010). It can also overactivate neuronal NADPH oxidase and mitochondrial xanthine oxidase. These effects reduce the antioxidant capacity of cells and contribute to ROS overproduction. Hyperglycemia-induced ROS react with cellular and/or circulating NO to form reactive nitrogen species (RNS) and peroxynitrite (Zochodne et al., 2000). RNS react with the thiol groups of SNAP proteins (Di Stasi et al., 2002), impairing the formation of neuronal regeneration cones. Consequently, RNS severely impact the capacity of neurons to repair themselves after oxidative damage (Kennedy and Zochodne, 2005). Currently, no therapy aims to reduce RNS generation, as it is still considered to be a contributing factor to DPN rather than an inducer. Please refer to Figures 2, 3 for visual summaries of NO interactions with neurons and glia.

### Advanced Glycation end Products

Advanced glycation end products (AGE) comprise a heterogeneous group of molecules formed by the non-enzymatic reaction of a sugar with an amino acid, a protein, a lipid, or a nucleic acid (Marieb et al., 2009). AGE precursors pass through several dehydration and redox reactions and molecular rearrangements to form AGEs (Sugimoto et al., 2008). The initial reaction leading to AGE formation is reversible, and depends on the available quantity of substrate (glucose) (Brownlee, 2005). However, in cases of chronic hyperglycemia (diabetes), AGE precursors are not degraded, but rather build in numbers, thereby generating AGEs (Sugimoto et al., 2008). As seen in Figure 2, AGEs are highly reactive, and can affect any type of protein, including matrix, basal membrane and structural proteins (Sugimoto et al., 2008). For example, AGEs bind to and modify the myelin of nervous fibers, prompting their phagocytosis by circulating macrophages or microglia (Bruck and Friede, 1990). This process contributes to classical DPN segmental demyelination (Said, 2007). AGEs can also directly interact with tubulin and actin neurofilaments found in neurons' axonal cytoskeletons (Sugimoto et al., 2008). AGE-directed modification of these proteins impairs axonal transport, and promotes axonal atrophy and/or degeneration (Sugimoto et al., 2008). The glycation of extracellular matrix (laminin) membranes is AGE-mediated and can counteract the innate ability of neurons to self-repair (Duran-Jimenez et al., 2009; Singh et al., 2014). AGE additionally binds to specific membrane receptors known as RAGE (Haslbeck et al., 2005), driving the transcription of pro-inflammatory mediators (Brownlee, 2005). RAGE stimulation increases matrix metalloproteinase production, which can further exacerbate nerve fiber damage (King, 2001). AGEs demonstrably accumulate in hyperglycemic patients experiencing retinopathy, nephropathy, hypertension and neuropathy (Brownlee, 2005). Environmental pollutants, smoking, and poor nutrition also enhance AGE formation (Sugimoto et al., 2008). Avoiding these factors can help control AGE formation and its associated damage (Sugimoto et al., 2008; Singh et al., 2014). Specific inhibitors such as aminoguanidine improve patients' nerve conduction velocity and neuronal blood flow, in addition to mitigating apoptosis and oxidative stress (Sugimoto et al., 2008; Orman et al., 2015).

### Ion Imbalance

#### Na^+^/K^+^ ATPase Pump

The Na^+^/K^+^ ATPase pump is a ubiquitous, energy-dependent enzyme implicated in the cellular membrane transport of ions. Using ATP, it transfers three sodium ions outside the cell in exchange for two potassium ions transported into the cell (Kumar et al., 2005). In doing so, it maintains the membrane's electric potential and nerve conductance (Creange et al., 2006). During hyperglycemia, impairments to polyol pathways (section Polyol Pathway) and PKC activity alter the function of the Na^+^/K^+^ ATPase pump leading to faulty nerve conduction (Creange et al., 2006). Reduced intracellular potassium levels curbed nodal potassium conductance, thereby affecting axonal excitability (Misawa et al., 2005). Hypokalemia can also alter Ca^2+^/K^+^ pump function, leading to neuronal hypocalcaemia.

#### Calcium

In early stages of DPN, the elevated levels of intracellular calcium disrupt nerve conductance (Kostyuk et al., 2001) and can, through calcium cytotoxicity, cause irreversible damage to the nerve fibers (Creange et al., 2006). Conversely, the hypocalcaemia observed in later stages of DPN mediates axonal degeneration, as seen in Figure 2 (Gispen and Hamers, 1994). Overall, impaired neurotrophic factor levels in DPN-afflicted neurons drive mitochondrial depolarization and Ca^2+^ concentration impairment, which in turn negatively impacts the TCA cycle and ATP production (Fernyhough and Calcutt, 2010). ATP dysregulation impairs cellular calcium homeostasis, reducing levels of endoplasmic reticulum (ER) and plasma membrane Ca^2+^ pumps (PMCA), as evidenced in streptozotocin (STZ)-treated rats. The impairment of ER calcium homeostasis disrupts protein synthesis, post-translational modification, and trafficking, all of which may contribute to distal axonal degeneration (Fernyhough and Calcutt, 2010). T-type calcium channel blockers notably improve thermal and mechanical hypersensitivity in T2D mice (Misawa et al., 2009).

#### Sodium

The DRG neurons of STZ-treated rats show an increased expression of sodium channels (Na_V_1.3, Na_V_1.6, and Na_V_1.9) (Craner et al., 2002; Hong et al., 2004), which contributes to ectopic impulse generation and neuronal hypersensitivity (Fischer and Waxman, 2010). The groups of Howe (Howe et al., 1977) and Wall (Wall and Gutnick, 1974) demonstrated that thermal, mechanical, and chemical stimuli thresholds are reduced following spontaneous electrical activity. Uninjured axons proximal to the affected neurons also exhibit ectopic discharge. Both of these phenomena result in increased electrical impulses in the spinal dorsal horn. The association between ectopic discharges and increased sodium channel expression can partly account for the therapeutic efficacy of anticonvulsant and tricyclic antidepressants in the treatment of DPN (Spruce et al., 2003).

### Polyol Pathway

Cellular glucose is converted into pyruvate by the actions of diverse enzymes implicated in glycolysis. In hyperglycemic conditions, excess glucose is not oxidized, but rather directed to the polyol pathway (Oates, 2002). Firstly, aldose reductase (AR) metabolizes glucose into sorbitol, which is later transformed into fructose by sorbitol dehydrogenase. Fructose is, notably, ten times more potent than glucose in generating AGE (Oka and Kato, 2001) (section Advanced Glycation End Products). Aldose reductase and sorbitol dehydrogenase are characterized by their lowered substrate affinity (elevated Km); the concentration of available substrate is therefore the limiting factor of this reaction (Oates, 2002).

Elevated sorbitol levels have been associated with cellular and organ damage (Oyama et al., 2006). It is believed that sorbitol directly depletes bioavailable myoinositol (MI) and increases its expulsion from the cell (Oka and Kato, 2001; Oates, 2002). Elevated blood sugar also prevents sorbitol's cellular reuptake by saturating its membrane transporter. A deficit in MI alters the metabolism of phospho-inositides, reducing diacylglycerol (DAG) and inositol triphosphate (IP_3_) production. This results in a lesser activation of PKC, which is itself a key activator of the Na^+^/K^+^ ATPase pump (Oka and Kato, 2001). A reduction in Na^+^/K^+^ ATPase pump activity triggers an intracellular reduction in K^+^, combined with increases in the concentration of Na^+^. In neurons, imbalances in ionic charges directly contribute to DPN by generating conductance anomalies (Oka and Kato, 2001). In this context, decreases in intracellular sodium concentrations affect sodium-dependent membrane transport. This transport is implicated in the reuptake of several amino acids and MI, contributing to a retro-positive feedback loop (Das Evcimen and King, 2007; Oates, 2008). Disruptions in the polyol pathway also increase ROS levels (section Reactive Oxygen Species) by reducing the production of glutathione, in addition to intracellular levels of antioxidants (NADPH). Polyol pathway pathologies also reduce the production of NO, which can in turn enhance vessel constriction. This impairs endothelial cell function can lead to the onset of microangiopathy (section Microangiopathy) (Oka and Kato, 2001); the effects of polyol pathways in DPN can be seen in Figure 1.

### Aldose Reductase

In patients with T1D, polymorphisms in the genes coding for aldose reductase (AR) can impair thermal nociceptive thresholds (Thamotharampillai et al., 2006). Higher AR levels also correlate with a higher severity of intra-epidermal nerve fiber loss (Hirai et al., 2000). AR specific inhibitors (ARi) have been shown to reverse or delay the onset of DPN in diabetic animals (Schemmel et al., 2009). While ARis are unavailable on the US market, they are currently being used clinically in Japan (Hotta et al., 1996). Epalrestat is currently the only commercially available inhibitor (Singh Grewal et al., 2016); and prevented the progression of diabetic neuropathy and retinopathy/nephropathy in neuropathic patients as compared to a control group (Hotta et al., 2012).

## Neuron Extrinsic Factors Promoting Diabetic Pain Neuropathy

Several neuron-extrinsic factors contribute to the onset and maintenance of neuropathic pain. Recent data has highlighted the key contribution of immune cells, acting as extrinsic factors, in driving DPN. Normally, the immune and sensory nervous systems work in concert to preserve homeostasis. They do so via interactions and exchanges between receptors, cytokines and neuropeptides (Talbot et al., 2016b; Veiga-Fernandes and Mucida, 2016). While this bidirectional communication helps to protect humans from danger, it can also contribute to disease pathophysiology (Chiu et al., 2013; Wilson et al., 2013; Talbot et al., 2015, 2016b; Foster et al., 2017). In fact, the somatosensory nervous system is anatomically positioned within primary and secondary lymphoid tissues and mucosa so as to interact with the cells of the immune system (Downing and Miyan, 2000; Rosas-Ballina et al., 2011; McMahon et al., 2015; Talbot et al., 2015; Veiga-Fernandes and Mucida, 2016; HD iPSC Consortium., 2017). Nociceptors, when sensing immunocyte-released cytokines, lower their firing thresholds; in doing so, they incite pain hypersensitivity (Wilson et al., 2013). While various immunocytes contribute to this phenomenon, this review will focus on the crucial role of microglia. Finally, we will review the role of blood vessels, which supply oxygen and glucose to the nerve, in generating DPN.

### Microglia

The central nervous system (CNS) is primarily composed of afferent and efferent neuron fibers that transport electrical signals to and from the periphery, oligodendrocytes that form and repair myelin, and astrocytes and microglia that support and protect neurons (Marieb et al., 2009). Microglial cells represent 10–15% of all cells found within the human brain (Foster et al., 2015). Glia act as resident macrophage-related cells of the CNS, comprising the first line of defense against pathogen invasion, generating innate immune responses by recognizing, sequestering and processing antigens (Lawson et al., 1992). While there are two major types of microglia: resident, and perivascular (Gosselin et al., 2010), which express receptors for most inflammatory neurotransmitters (Hickey and Kimura, 1988), it seems that glia exists in nine distinct subtypes with different functions, appearance, and presence. Resident microglial cells are bone marrow-derived hematopoietic cells that invade the CNS during embryonic development (Pocock and Kettenmann, 2007). These are very rarely replaced, and rapidly proliferate while activated (Milligan and Watkins, 2009). Conversely, perivascular microglial cells are continuously replenished by bone marrow-derived hematopoietic precursors (Gosselin et al., 2010), particularly during CNS inflammation (Romero-Sandoval et al., 2008). Perivascular microglia can alter the blood-brain barrier's permeability, and exert anti-inflammatory effects, while resident microglial cells exert both pro- and anti-inflammatory effects (Milligan and Watkins, 2009).

While on a polarization continuum, microglia activation can be classified as either resting or activated. A resting microglial cell possesses a small soma with thin and ramified processes (Tsuda et al., 2005), express immunoreceptors (Lawson et al., 1992), and perform immune roles to maintain CNS homeostasis (Tsuda et al., 2005). Upon activation due to trauma, inflammation, or infection, microglia undergo several stereotypic changes in morphology, gene expression, function, and number (Tsuda et al., 2005). They upregulate various transmitters and receptors, including the complement receptor 3 (CR3) (Eriksson et al., 1993; Lassmann et al., 1993), major histocompatibility complex 2 (MHC2) (Shi et al., 2017), TLR4 (Sweitzer et al., 2002), and CD14 (Sweitzer et al., 2002). The intracellular events promoting glial cell activation remain unclear, but are known to involve the activation of cannabinoid CB2 receptors (Tanga et al., 2004), kinin B1 receptors (Li and Kim, 2017), P_2_X_4_R (Noda et al., 2007), NK-1R (Inoue, 2008), CX3CR-1 (Abbadie et al., 2009; Gao and Ji, 2010; Zhou et al., 2010), CCR-2 (Zhang and De Koninck, 2006; Milligan et al., 2008), MMP9 (Thacker et al., 2009), BDNF-R (Kawasaki et al., 2008), TLR3 (Tender et al., 2010) and TLR4 (Kim et al., 2007); leading to the phosphorylation of p38 MAPK (Jin et al., 2003; Tsuda et al., 2004; Tanga et al., 2005; Daulhac et al., 2006; Ji et al., 2009). For more information on microglia intracellular signaling refer to Popiolek-Barczyk and Mika (Chang et al., 2010). Furthermore, microglial activation can occur via endogenous pro-inflammatory signals (IL-1β, TNFα, IL-6, and NO), opioids (Popiolek-Barczyk and Mika, 2016) or heat shock protein (Hutchinson et al., 2008). Activated microglial soma increase in size and their long and thin ramifications withdraw, ultimately resulting in an amoeboid shape with few ramifications (Tsuda et al., 2005). They have implicated microglia in reward behavior (Costigan et al., 1998) as well as in the onset of chronic neurodegenerative diseases (Taylor et al., 2015; Hammond et al., 2018), such as lupus erythematosus (Salter and Stevens, 2017), Huntington's chorea (Nestor et al., 2018), and Alzheimer's disease (Eriksson et al., 1993).

### Painful Glia

Microglial cells produce and release various excitatory peptides, including PG, SP, EAA, NO, and ATP, and express selective receptors for immunomodulatory neurotransmitters. Both peptides and receptors allow microglia to detect, and respond to, neuronal signals, thereby generating autocrine or paracrine feed forward inflammatory loops (McMahon et al., 2005). For example, TNFα and MMPs activate microglial p38 MAPK in the spinal cord dorsal horn during peripheral neuropathic pain (Svensson et al., 2005). MMP9-induced pro-IL-1β cleavage leads to p38 MAPK phosphorylation in microglia during the onset and early stages of neuropathic pain. MMP2-induced pro-IL-1β cleavage leads to astrocyte activation in later disease stages (Kawasaki et al., 2008). ATP-stimulated microglial P_2_X_4_R enhances the levels of intracellular Ca^2+^. Such influx activates various transcription factors, including NF-κB, p38, and ERK-MAPK (Tsuda et al., 2005), leading to the synthesis of pro-inflammatory cytokines (IL-1β, TNFα, and IL-6) (Watkins et al., 2001a; Marchand et al., 2005) or neuroexcitatory substances such as D-serine (Petrenko et al., 2003). This transcriptomic profile can initiate and maintain neuropathic pain by facilitating neuron-glial interactions (Hickey and Kimura, 1988; Abbadie et al., 2009). The interplay between neurons and glia can therefore sustain neuronal stimulation and sensitization by increasing glutamatergic stimulation and by reducing GABAergic inhibitory signals (Tsuda et al., 2005; Inoue, 2006; Scholz and Woolf, 2007; Biggs et al., 2010); the process can be seen in Figure 3.

In current pre-clinical literature, activated microglia have emerged as key drivers of pathological pain in chemotherapy-induced neuropathy and peripheral nerve and spinal cord injuries (Watkins et al., 2001b; Marchand et al., 2005; Tsuda et al., 2005; Daulhac et al., 2006; Pocock and Kettenmann, 2007; Gadani et al., 2015; McMahon et al., 2015). For example, CB2 receptor agonists dramatically attenuate iNOS induction and ROS generation in LPS-activated microglia (Ribeiro et al., 2013). The incitement of inflammation signals microglia to migrate, proliferate, synthesize and release pro-inflammatory mediators that maintain neuron activation. In keeping with the fact that an intrathecal injection of activated microglia induces both thermal hyperalgesia and tactile allodynia (Tsuda et al., 2003; Narita et al., 2006), while resting microglia or activated astrocytes are without effect (Narita et al., 2006). Blockades of p38 MAPK (Tanga et al., 2005; Ji et al., 2009), CX3CR-1 (Milligan et al., 2004; Verge et al., 2004; Sun et al., 2007) or P_2_X_4_R (Tsuda et al., 2003) have been shown to alleviate chronic pain in rodents. Other chemokines, such as RANTES, IP-10, and SDF1 are also implicated in enhanced microglia migration, infiltration, phagocytosis; they are therefore contributors to microglia-induced neuropathic pain (White et al., 2007).

Salter and Beggs have made several discoveries linking neuronal hypersensitivity to overactive microglia (Beggs and Salter, 2016). Firstly, they were able to show that nerve injury activates microglia and causes them to express P_2_X_4_R. They demonstrated that the IRF-8/IRF-5 transcriptional cascade clearly regulates the expression of P_2_X_4_R gene. Additionally, they showed that external stimulation (CCL2 and LPS) leads to the translocation of P_2_X_4_R protein from lysosome to cell surface (Tsuda and Inoue, 2016). A targeted silencing of P_2_X_4_R suppressed injury-induced tactile allodynia, while an intraspinal administration of P_2_X_4_R-expressing glia had the opposite effect (Tsuda et al., 2003). They also identified spinal dorsal horn neurons as a source of ATP (Masuda et al., 2016), and that ATP-stimulated microglia release brain derived neurotrophic factor (BDNF). BDNF limits GABAergic inhibitory signals sent to afferent nociceptor neurons (Torsney and MacDermott, 2005); this enhances their activity by uncoupling the transmembrane anion gradient (Coull et al., 2005). A disruption of chloride transport changed spinal lamina I neurons' phenotype, causing them to (i) increase nociceptive responsiveness, (ii) relay innocuous mechanical inputs, and (iii) generate spontaneous bursts of activity; respectively, accounting for (a) hyperalgesia, (b) mechanical allodynia, and (c) spontaneous pain (Keller et al., 2007). The microglia-to-neuron P_2_X_4_R -BDNF-KCC2 axis was also found to drive opioid-induced thermal hyperalgesia. Interestingly, Salter and Beggs found that this hyperalgesia mechanistically differs from opioid-induced tolerance (Beggs and Salter, 2016). Microglia-derived BDNF also emerged in their research as a negative regulator of reward in opioid-dependent states (Taylor et al., 2016), while the Panx1-mediated release of microglial ATP controls morphine withdrawal without affecting opiate-induced analgesia (Burma et al., 2017).

Peripheral tissue injury was found to increase the intensity, spatial distribution, and persistence of Iba-1^+^ microglial activity within the spinal dorsal horn, resulting in a long-lasting priming of withdrawal reflex sensitivity and microglial responsiveness (Beggs et al., 2012). MCP-1^+^ neurons drive the bone marrow-derived microglial infiltration of injured spinal cords. These neurons also facilitate glial activation and drive mechanical allodynia (Zhang et al., 2007). CCL21, a microglial activator, is also found to be upregulated in the cell bodies of spinothalamic tract neurons following nerve injury. This molecule is transported rostrally to the thalamus, where it activates microglia and drives neuron hyperexcitability (Zhao et al., 2007b). Spinal microglia consequently remain activated for more than 3 months following nerve injury in rodents, thereby maintaining chronic pain (Echeverry et al., 2017). Newly-generated microglia also appear to be coded with an inherent “memory” of previous injuries, contributing to long-lasting neuropathic pain (Yao et al., 2016).

Following a spinal cord injury, microglial ERK kinases are activated, prompting the intraspinal release of PGE_2_, which results in spinal cord dorsal horn neuron hyperresponsiveness (Zhao et al., 2007a). Following the spinal release of IL-1β and IL-6, fractalkine triggers pain by activating microglia expressing CX3CR1 (Milligan et al., 2004, 2005). IL-1β upregulates both neuronal NMDAR phosphorylation and expression. These effects enhance NMDAR conductivity (Zhang et al., 2008) and calcium influx (Viviani et al., 2003), increasing neuronal excitability and synaptic strength (Beattie et al., 2002; Stellwagen and Malenka, 2006). Microglial NO and PGE_2_ can also increase the excitability of pain-projecting neurons (Besson, 1999). Overall, a modulation of microglial polarization was shown to alleviate neuropathic pain (Chang et al., 2010; Piotrowska et al., 2016; Xu et al., 2018). Intriguingly, microglia only induce mechanical pain hypersensitivity in male rodents (Mapplebeck et al., 2018), as T-lymphocytes appear to be responsible for this mechanism in females (Sorge et al., 2015).

### Microglia, an Emerging Target in DPN

Given that db/db mice and T2D patients show increased levels of activated microglia (Arroba and Valverde, 2017), and that the pharmacological inhibition of spinal resident microglia reverses painful neuropathy (Wodarski et al., 2009; Sun et al., 2015; Lin, 2017; Zhang et al., 2018), we take the overall view that microglia may drive DPN (Figure 1). Activated microglia levels have been found to correlate with thalamus hyperresponsiveness (Fischer and Waxman, 2010), and the brain thalami of DPN patients demonstrate increases in blood flow (Paulson et al., 2007), spontaneous neuronal activity (Fischer et al., 2009), receptive field size enhancement (Fischer et al., 2009), and alterations in neuronal connectivity (Cauda et al., 2010). Additionally, activated microglia drive DPN in STZ-treated rats (Wodarski et al., 2009; Talbot et al., 2010); this process can successfully be reversed by gabapentin (Wodarski et al., 2009) or minocycline treatment (Talbot et al., 2010).

Additionally, diabetes-induced hyperglycemia enhances microglial NADPH oxidase and iNOS activation (Figure 3), promoting the production of ROS (Quan et al., 2007, 2011) and peroxynitrite (Li et al., 2005). As a result of the iNOS-NO-NRS axis, activated microglia are a major source of free radicals in the spinal cords of animals with DPN (Li et al., 2005; Candelario-Jalil et al., 2007). Glial-released NO inhibits neuronal cytochrome oxidase, blocking mitochondrial respiration, which in turn depletes the production of ATP (Brown and Bal-Price, 2003). Moreover, ROS and RNS deplete endothelial cell NO levels, contributing to the generation of microangiopathy (section Microangiopathy). A reduction in neuronal blood supply leads to hypoxia and mitochondrial dysfunction; this results in cellular energy deficits, and ultimately, neuron death. Finally, elevated ROS levels also impair axonal transport (Larsen and Sidenius, 1989) and the capacity of the nerve to repair itself (Longo et al., 1986), further worsening nerve health in DPN patients.

Microglia exposed to high glucose levels show increases in mRNA expression, and secrete TNFα and MCP-1, leading to neuronal activation (Quan et al., 2011). The spinal microglia's activation of P2Y12 and P2Y13 receptors triggers the production of IL-1β and IL-6 in a rat model of DPN (Liu et al., 2017; Zhou et al., 2018). The activation of microglial RAGE (Thornalley, 1998) leads to the release of chemokines CCL3, CCL5, and CXCL12, which activate microglia (Bianchi et al., 2011). The resulting activated microglia actively phagocyte neuronal myelin, thereby promoting DPN (Mosley and Cuzner, 1996).

### Microangiopathy

Microangiopathy is characterized by the shrinking and weakening of small blood vessels. This pathology leads to a reduction in blood flow, protein leakage, and bleeding. In diabetic states, endothelial cells take up excessively large quantities of circulating glucose, raising the production of AGE (section Advanced Glycation End Products). These AGEs enhance the proliferation of endothelial cells, which leads to a thickening of basal membranes and progressive vessel occlusion, which are associated with reduced neuronal blood flow (Tuck et al., 1984; Cameron et al., 1991) and ischemia. In turn, the ischemia reduces neuronal oxygen and nutrient supplies, triggering nerve fiber loss (Yagihashi et al., 2007). Microangiopathy in neuron-irrigating vessels also impairs their capacity to regenerate and repair (Kennedy and Zochodne, 2005). A thickening of basement membranes and endothelial cell swelling thereby positively correlates with reduced nerve fiber myelin densities (Yagihashi et al., 2007). The occlusion of neuronal capillaries promotes axonal degeneration and a dysfunctional loss of sensory perception, as typically observed in the later stages of DPN.

Multifocal nerve lesions and alterations in endoneurial capillaries indicate a role for circulatory factors in the symmetrical form of DPN (Dyck et al., 1986; King et al., 1989). Peripheral nerve fiber loss in DPN is well-associated with the increased migration and infiltration of inflammatory cytokines released by T-lymphocytes and macrophages (Said et al., 2003; Said, 2007). These cytokines increase vessel damage and promote microangiopathy (Said, 2007). Considering that microglia and macrophages share similar roles and inflammatory characteristics, it is probable that microglia may also promote microangiopathy within the CNS (Figure 3).

## Conclusion and Future Therapeutic Directions

Multiple molecular mechanisms generate and amplify hyperglycemia-induced neuropathic pain. This collaboration involves voltage-gated ion channels, ligand-gated channels, cytokine receptors, direct myelin damage (Edwards et al., 2008; Vincent et al., 2008), neuronal depolarization (Abbadie et al., 2009), conduction impairment (Kramer et al., 2004), a loss of interneuron inhibitory input (Wood, 2008), Aβ-fiber sprouting (Yasuda et al., 2003), and neuronal death (Inoue, 2006). This multicentric view accounts for why hyperglycemia-induced DPN remains highly refractory to treatment paradigms. Given such potential heterogeneity amongst patients, personalizing of DPN management may prove useful. This will necessitate improved diagnostic methods and personalized medicine tailored to the specific pathology in question.

While design, safety and drug efficacy often vary between rodent and clinical models of disease, calcium channel α2-delta ligands (pregabalin, gabapentin), antidepressants (TCA and SSRI), and opioid-like drugs as well as topical agents including capsaicin, lidocaine, or botulinum toxin A can help alleviate patients' DPN (Finnerup et al., 2015). The long-term efficacies of these approaches have yet to be demonstrated. Overall, the high plasticity of microglial phenotypic and transcriptomic changes persists even after glycemic control, inciting and maintaining neuron sensitization (Milligan et al., 2008). Therefore, the microglia, which potentially act upstream of pain neurons, should rightly be considered as a crucial therapeutic target in the treatment of diabetic pain neuropathy. Future therapies may therefore involve targeting specific receptors and signaling cascades which engage such deleterious neuro-immune crosstalk.

## Author Contributions

TR, RC, AC, and ST conceived the study and wrote the manuscript while SCT, J-CW, MA, MB, TC, and JD contributed to its design.

### Conflict of Interest Statement

The authors declare that the research was conducted in the absence of any commercial or financial relationships that could be construed as a potential conflict of interest.
